# Mutated *VWA8* Is Associated With Developmental Delay, Microcephaly, and Scoliosis and Plays a Novel Role in Early Development and Skeletal Morphogenesis in Zebrafish

**DOI:** 10.3389/fcell.2021.736960

**Published:** 2021-10-01

**Authors:** Muhammad Umair, Muhammad Farooq Khan, Mohammed Aldrees, Marwan Nashabat, Kheloud M. Alhamoudi, Muhammad Bilal, Yusra Alyafee, Abeer Al Tuwaijri, Manar Aldarwish, Ahmed Al-Rumayyan, Hamad Alkhalaf, Mohammad A. M. Wadaan, Majid Alfadhel

**Affiliations:** ^1^Medical Genomics Research Department, King Abdullah International Medical Research Center (KAIMRC), Ministry of National Guard Health Affairs (MNGH), King Saud Bin Abdulaziz University for Health Sciences, Riyadh, Saudi Arabia; ^2^Bioproducts Research Chair, Department of Zoology, College of Science, King Saud University, Riyadh, Saudi Arabia; ^3^Division of Genetics, Department of Pediatrics, King Abdullah Specialized Children’s Hospital, King Abdulaziz Medical City, Riyadh, Saudi Arabia; ^4^Department of Molecular Oncology, King Faisal Specialist Hospital and Research Center, Riyadh, Saudi Arabia; ^5^Department of Biochemistry, Faculty of Biological Sciences, Quaid-i-Azam University, Islamabad, Pakistan; ^6^Pediatric Neurology Division, Department of Pediatrics, Ministry of National Guard-Health Affairs (MNGHA), King Saud Bin Abdulaziz University for Health Sciences, Riyadh, Saudi Arabia; ^7^Department of Pediatrics, Ministry of National Guard-Health Affairs (MNGHA), King Saud Bin Abdulaziz University for Health Sciences, Riyadh, Saudi Arabia; ^8^Genetics and Precision Medicine Department (GPM), Ministry of National Guard Health Affairs (MNG-HA), King Abdullah Specialized Children’s Hospital (KASCH), King Abdulaziz Medical City, Riyadh, Saudi Arabia

**Keywords:** *VWA8*, whole-exome sequencing, novel gene, missense variant, zebrafish morpholino

## Abstract

Von Willebrand A domain-containing protein 8 (*VWA8*), also named KIAA0564, is a poorly characterized, mitochondrial matrix-targeted protein having a putative ATPase activity. *VWA8* is comprising of ATPase-associated domains and a VWFA domain associated with ATPase activity inside the cell. In the present study, we describe a large consanguineous family of Saudi origin segregating a complex developmental syndrome in an autosomal recessive fashion. All the affected individuals exhibited severe developmental disorders. DNA from three patients was subjected to whole-exome sequencing followed by Sanger sequencing. *VWA8* knock-down zebrafish morpholinos were used to study the phenotypic effect of this gene on zebrafish development. A homozygous missense variant [c.947A > G; p.(Asp316Gly)] was identified in exon 8 of the *VWA8* gene, which perfectly segregated with the disease phenotype. Using zebrafish morpholino, we observed delayed development at an early stage, lack of movement, light sensitivity, severe skeletal deformity such as scoliosis, and facial dysmorphism. This is the first homozygous variant identified in the *VWA8* gene underlying global developmental delay, microcephaly, scoliosis, limbs, and cardiovascular malformations in humans. We provide genetic and molecular evidence using zebrafish morpholino for a homozygous variant in the *VWA8* gene, associated with such a complex developmental syndrome in humans.

## Introduction

The human *VWA8* gene, also named *KIAA0564*, is localized to chromosome 13, while the zebrafish *vwa8* gene is located on chromosome 09. VWA8 protein consists of two isoforms, the long (VWA8a) and the short isoform (VWA8b). It is named von Willebrand factor A domain containing 8 (Vwa8) due to the C-terminus location of von Willebrand Factor type A (vWFA). The vWFA domains are highly conserved across eukaryotic species, associated with membrane and ribosomal transport and involved in different protein-protein interactions concomitant with multi-protein complexes (Whittaker and Hynes, 2002).

The *VWA8* longest isoform consists of 45 exons, which encodes a protein of 1905 amino acids with assumed ATPase activity (Luo et al., 2017). The VWA8 expression appears to vary among different transcripts and shows different expression levels in cDNA clones from the hypothalamus amygdala, substantia nigra, and whole brain, etc. The associated variant might also regulate nearby other genes (Oedegaard et al., 2010).

Studies suggested that genome-wide linkage analysis of 13 pedigrees with bipolar affective disorder showed susceptibility to chromosome 13q14 [marker D13S153 (LOD score: 2.29)], which is located close to *KIAA0564* (Badenhop et al., 2002). VWA8 (KIAA0564) also showed putative ATPase activity expressed in the brain, as seen in patients with familial hemiplegic migraine (FHM II), where *ATP1A2* is mutated, which encodes for two subunits of Na+/K+ ATPases, expressed in the adult brain (De Fusco et al., 2003). In addition, the chromosome 13q14.1 region harboring the *VWA8* gene has been associated with migraine headaches in patients with ADHD and BPAD (Oedegaard et al., 2010).

Although VWA8 protein is essentially uncharacterized, the human and zebrafish VWA8 amino acid sequences are 82–84% identical for the long and short isoforms. The VWA8 protein is primarily expressed in the kidney, liver, pancreas, skeletal muscles, heart, and skeletal muscles (Whittaker and Hynes, 2002).

In the present study, we presented a large Saudi consanguineous family segregating severe developmental disorder in an autosomal recessive fashion. WES identified a homozygous missense variant in the *VWA8* gene located at chromosome 13q14.11.

## Materials and Methods

### Study Approval

**FIGURE 1:**
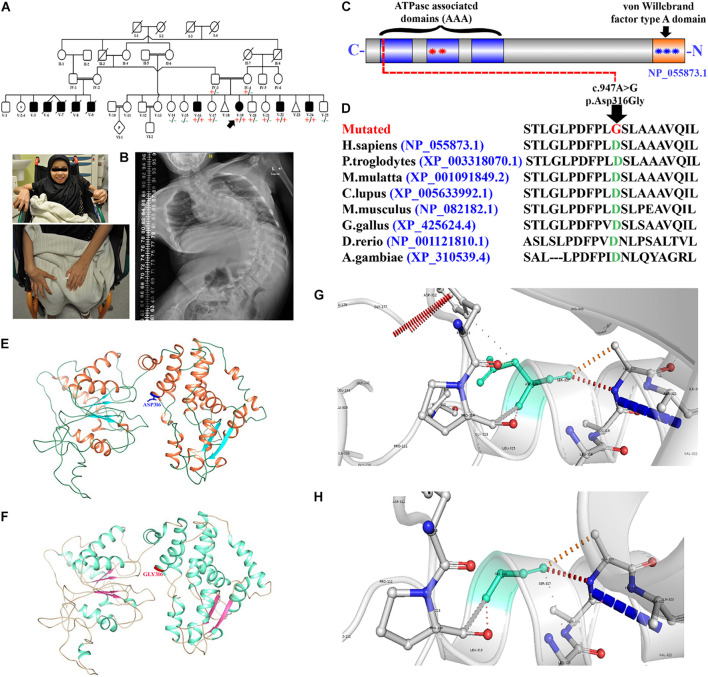
Pedigree and molecular analysis. **(A)** Pedigree clearly depicts the autosomal recessive mode of inheritance. Squares and circles represent males and females. White symbols represent normal, while black symbols represent affected individuals, respectively. The double line represents the consanguineous union. **(B)** Picture of the affected individual (V-19) showing developmental delay and bound to a wheelchair. Posteroanterior spinal radiographs demonstrating severe scoliosis. **(C)** Schematic representation of the VWA8 domains (1905 amino acids). Each contains three predicted ATPase, dynein-related AAA domains (blue) with the second AAA domain-containing an ATPase binding site bounded by the Walker A (first red star) and Walker B (second red star) motifs. VWA8a has a predicted von Willebrand factor type A domain (blue star) at the C-terminus containing a metal ion-dependent adhesion site (MIDAS). **(D)** Representing partial amino acid sequence of the VWA8 amino acid acids, depicting the conservation of Asp316 amino acid across different species. **(E,F)** Structural 3D representation of VWA8^Asp316^ (wild type) and VWA8^Gly316^ (mutated). **(G,H)** Zoomed cartonic representation of the wild type (VWA8^Asp316^) and mutated (VWA8^Gly316^) protein structure, showing changes in the bonding and overall structure.

Fresh blood samples were collected from all the affected individuals and normal family members (Figure 1A). The present study was approved by the Institutional Review Board (KAIMRC) and followed Helsinki protocols. Written informed consent for publication of this report, patient images, and clinical data was attained from the parents. The studies involving human participants were reviewed and approved by KAIMRC. Written informed consent was obtained from the individual(s), and minor(s) legal guardian/next of kin, for the publication of any potentially identifiable images or data included in this article.

### Clinical Evaluation of the Patient’s Phenotype

After the patient’s recruitment, routine laboratory tests were performed for all the four affected individuals (V-16, V-19, V-22, and V-24). These include growth parameters [height, weights, and head circumference], and brain MRI, skeletal survey (X-rays), hearing test, ophthalmic examination, and echocardiography (Table 1).

**TABLE 1:** Clinical description of affected individuals.

Clinical phenotypes	Individual 1 [V-19]	Individual 2 [V-16]	Individual 3 [V-22]	Individual 4 [V-24]
Sex	Female	Male	Male	Male
Origin	Saudi	Saudi	Saudi	Saudi
Consanguinity	+	+	+	+
Pregnancy event	Uneventful full term	Uneventful full term	Uneventful full term	Uneventful full term
Global developmental delay	+	+	+	+
Speech delay	+	+	+	+
Mild-moderate Intellectual disability	+	+	+	+
Age at last exam (year)	15	10	8	19
Head circumference	51 cm (<3rd percentile)	51 cm (<3rd percentile)	51 cm (10th percentile)	52 cm (<3rd)
Height	105 cm (<3rd percentile [–8.7 SD])	125 cm (<3rd percentile [–21.5 SD])	106 cm (<3rd percentile [–4.1 SD])	153 (<3rd)
Weight	30 kg (<3rd percentile [–3.3 SD])	20.8 kg (<3rd percentile [–2.6 SD])	19 kg (<3rd percentile [–2.4 SD])	34 kg (<3rd percentile)
Dysmorphic features	–	–	–	–
MRI brain	Normal	Normal	Normal	Normal
Skeletal survey	Severe thoracic and lumber scoliosis	Bilateral coxa magna and valga deformities with partial acetabular uncovering of femoral heads	Severe thoracic and lumber scoliosis	Severe thoracic and lumber scoliosis Bilateral club foot deformity
Hearing test	Normal	Normal	Normal	Normal
Eye exam	Astigmatism	Extropia, myopic astigmatism and amblyopia	Extropia, myopic astigmatism and amblyopia	Astigmatism
Echocardiogram	Large atrial septal defect	Normal	Normal	Normal
Genetic results	c.947A > G [p.(Asp316Gly)] in *VWA8* gene	c.947A > G [p.(Asp316Gly)]in *VWA8* gene	c.947A > G [p.(Asp316Gly)]in *VWA8* gene	c.947A > G [p.(Asp316Gly)]in *VWA8* gene

### Genomic DNA Extraction

Genomic DNA (gDNA) was extracted from all the family members’ fresh blood using a QIAampDNA Micro kit using standard procedures (Figure 1A). The quantification of the gDNA was assessed using a NanoDrop^TM^ spectrophotometer using traditional methods.

### Whole-Exome Sequencing

DNA of three affected individuals was subjected to WES using Ion Torrent (Ion AmpliSeq^TM^ Exome RDY kit PIv3, Rev. A.0; MAN0010084; Thermo Fisher Scientific, Inc.) platform CENTOGENE (Germany). Using Exome Primer Pools, the genomic DNA was amplified in separate wells *via* AmpliSeq HiFi mix (Thermo Fisher Scientific). The obtained PCR pools were combined in a single well and incubated with FuPa reagent (Thermo Fisher Scientific). The amplified exome targets were ligated with Ion Xpress and Ion P1 barcode adapters using standard procedure in the next step. Next, purification was performed, and the libraries were quantified using the Ion Library Quantification Kit (Thermo Fisher Scientific). Subsequently, emulsion PCR was performed using an Ion OneTouch System, and the template Ion Sphere particles were enriched using Ion OneTouch according to the manufacturer’s instructions. Finally, the template-positive Ion PI Ion Sphere particles were processed for sequencing. WES covered approx. 37 Mb of end-to-end tiled probe space. All the reads were aligned against human assembly hg19 (GRCh37^1^), and variants were identified using the Saudi Human Genome Program (SHGP) pipeline. Subsequently, filtering of different variants was performed using standard methods.

### Variant Filtration Steps

**FIGURE 2:**
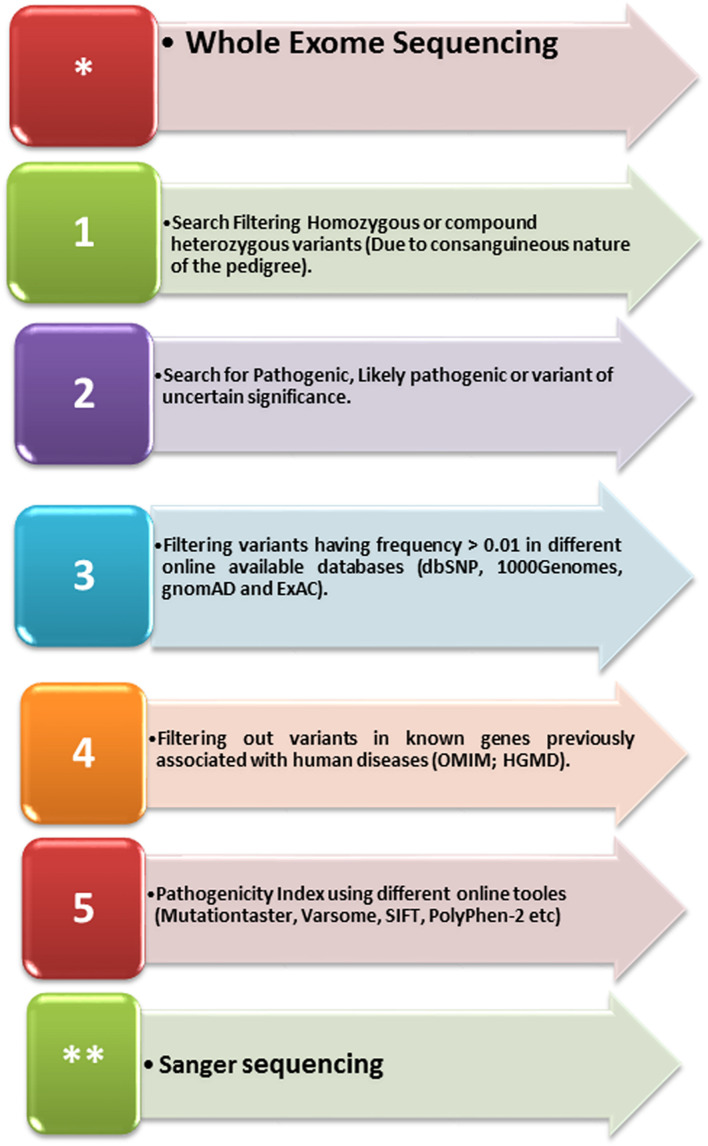
A schematic representation of the variant filtering and identification steps used in the present study.

As the pedigree depicted an autosomal recessive inheritance pattern, we searched for homozygous and compound heterozygous variants that were common among the affected individuals. Variants were filtered using the basic ACMG criteria such as (i) pathogenic, (ii) likely pathogenic, and (iii) variant of uncertain significance, while likely benign and benign variants were excluded. In addition, MAF of 0.01 was used to filter the variants and focused on homozygous variants that were not present in different online databases such as gnomAD, ExAC, dbSNP, and 1000 genomes. It was made sure that the identified variant has not been previously associated with any disease phenot ype using OMIM and HGMD. The pathogenicity of the identified variants was cross-checked using several online available tools such as Varsome, Mutationtaster, and SIFT (Table 2 and Figure 2).

**TABLE 2:** Pathogenicity index of the identified *VWA8* variant [c.947A > G; p.(Asp316Gly)] in the present study.

Prediction tool	Pathogenicity	Score	Range
PROVEAN	Damaging	–5.39, –4.99	–14 to +14
MutationTaster	Disease causing	1	–
SIFT	Tolerated, damaging	0.092, 0.026	0–1
Varsome	VUS	PP3, BP1	–
DANN	Pathogenic	0.9958	0–1
Mutation assessor	Medium	3.005	–5.135 to +6.49
FATHMM-XF	Damaging	0.8809	0–1
FATHMM-MKL	Damaging	0.9809	0–1
BayesDel addAF	Damaging	0.0892	–
LRT	Conserved	9.9999e–7	0–1
EIGEN PC	Pathogenic	0.4296	–
GERP	Conserved	5.5599	–12.3 to 6.17

### Sanger Sequencing

Once the variants were filtered, bi-directional Sanger sequencing was performed for all the members of the family. Bi-directional sequencing was performed according to the accredited protocol [Saudi Diagnostic laboratories, Riyadh, Saudi Arabia (Umair et al., 2016). Primers sequences were designed using Primer3 online software^2^ and will be provided upon request.

### *In Silico* Analysis

The identified variant’s pathogenicity index was calculated using different online available tools such as PROVEAN, Mutation Taster, SIFT, Varsome, Mutation Assessor, DANN, BayesDel added, and FATHMM-XF (Table 2). The frequency of the identified variant in the general population was calculated using ExAC/gnomAD,^3^ EVS,^4^ and 1000 genome project.^5^

### Protein 3D Modeling

The partial amino acid (540 amino acids) sequence of VWA8 encoding protein was retrieved from the UniProt database with accession number A3KMH1 in FASTA format. In the absence of an experimentally known structure, comparative modeling is one of the most precise computational approaches to predict a consistent 3D design from sequence Information (Källberg et al., 2012). Due to the absence of an experimentally known structure for VWA8, its protein sequence was submitted to the I-TASSER server for structure prediction (Yang et al., 2015). From models generated by I-TASSER, the model was selected based on the I-TASSER evaluation score. Multiple sequence alignment (MSA) of both protein sequences was done through CLUSTLW.^6^ The obtained 3D structures were subjected to energy minimization through UCSF Chimera version 1.5.6 (Meng et al., 2006) by 1,000 steps of steepest-decent (Wardi, 1988), followed by 1,000 steps of conjugate-gradient (Dai, 2000) minimization through AMBERff14SB force field. The stereo-chemical properties and Ramachandran values were assessed by the MolProbity server (Chen et al., 2010). Finally, Ramachandran outliers and poor rotamers were removed through WinCoot (Emsley et al., 2010) to obtain optimized and reliable structures for further computational analysis.

### Zebrafish Care and Husbandry

Zebrafish (Danio rerio) wild-type strain AB (Catalog ID: ZL1438) were obtained from zebrafish international resource center and raised in animal facility Bioproducts research chair, Department of Zoology, King Saud University, Riyadh, Saudi Arabia. The fish were maintained following the national and international guidelines for the laboratory animals’ care and use.

### Zebrafish Embryos

The fertilized embryos were obtained by natural pairwise breeding of adults. The fertilized embryos were sorted, and 1–2 cell stage embryos were used for microinjection.

### Ethical Approval

All the embryos used in this study were less than 5 days post-fertilization. Hence, they were exempted from taking the ethical committee’s approval for laboratory animals’ use and care (Strähle et al., 2012).

### Zebrafish Microinjection and Design of Morpholino Antisense Oligos

Morpholinoes (MOs) were purchased from Gene Tools (Philomatch, United States) and diluted in sterile water at the concentration of 1 mM. Two antisense oligo morpholinos were designed to target exon 2 of zebrafish *vwa8*. The i1e2 oligo targets the intron 1/exon 2 splice junction, the acceptor splice site of exon 2. The e2i2 oligo targets the exon 2/intron 2 splice site, the donor splice site of exon 2. Morpholino oligo sequence written from 5′ to 3′ and complementary to i1e2 splice junction target is “ACAGTGTCACCTGTGAAGAAAACGA,” I1e2 5bp mismatch control “GTaTaATAAgTTcCACATACTcTGA.” Morpholino oligo sequence written from 5′ to 3′ and complementary to E2i2: “TTTTATAACTTGCACATACTGTGA,” and E2i2 5bp mismatch control “ACAcTcTCACCTcTGAAcAAAACcA.” For single knockdown, i1e2 MO used is 3 ng/embryo, and e2i2 MO was 6–12 ng/embryo. Both morpholino was injected together with keeping i1e2 MO as 1 ng/e2i2 MO was 5 ng/embryo.

### Confirmation of Morpholino Based Knockdown in Zebrafish Using Real-Time RT PCR

**FIGURE 3:**
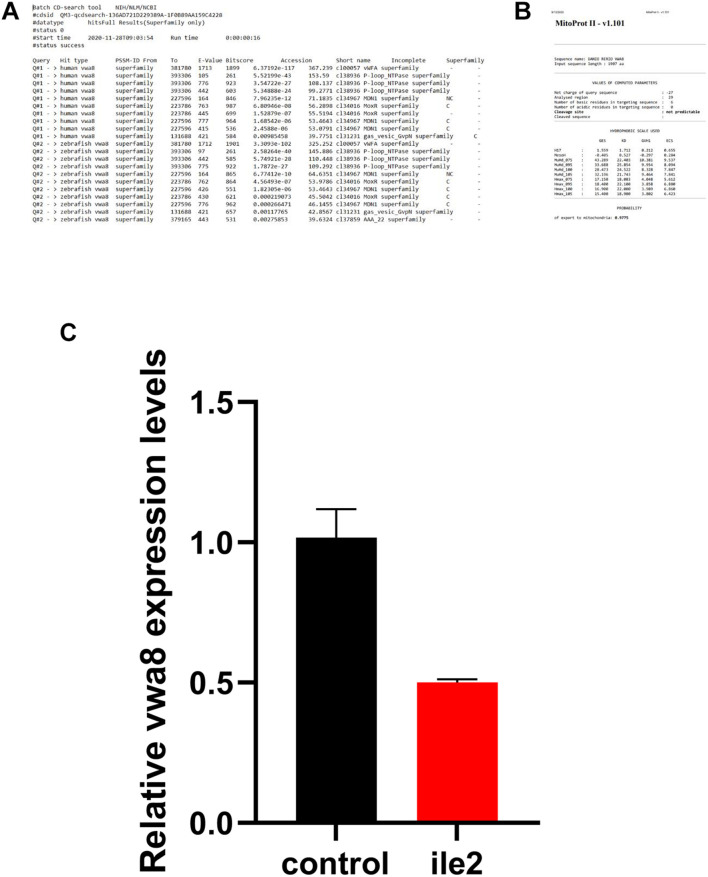
**(A)** Human and zebrafish-conserved domain search result. **(B)** The mitochondrial localization signal. **(C)** The knockdown efficiency of each morpholino was verified by real time RT-PCR at 72 hpf. The results revealed that the antisense morpholino showed substantially reduced expression as compared to the 5 bp match control.

In order to check the knockdown efficiency of *vwa8* in morpholino injected embryos (72 h), real time RT PCR was conducted. As the morpholinos were designed to target the splice junction of exon 2, RT PCR primers were designed flanking the exon 2 of zebrafish wva8 genomic sequence. The primer sequence is Forward 5′AACCCCCAAAAATCCAGAAC-3′ and Reverse 5′-AACGTGGCTTGTGCATAAAA-3′. Beta actin (same as above) was used for normalization, PCR was conducted as described above, and GraphPad Prism (version 8.1) was used to analyze the q-PCR results. A one-way ANOVA statistical test was applied, and a *p* < 0.05 value was considered significant (Figure 3C).

## Results

### Clinical Assessment

The proband (V-19) is a 10-year-old female with a global developmental delay. She was a full-term baby girl born as a result of a standard spontaneous vaginal delivery. Her birth weight was 3.2 kg (25th percentile), length: 48 cm (25th percentile), and head circumference: 35 cm (50th), APGAR score, 9 and 10 at 5 and 10 min, respectively. After delivery, she was discharged the next day in good condition.

The parents’ first concern was at 2 months of age when she started to develop shortness of breath and difficulty in feeding. At the age of 1 year, the parents noticed that she could not sit without support and was unable to walk. She was referred to genetics service at the age of 13 years for further evaluation where her examination showed growth parameters as follows: weight 21 kg (<1 percentile; –6.6 SD), length: 108 cm (<1 percentile; –21.0 SD), and head circumference: 50.5 cm (1 percentile; –2.4 SD) with no apparent dysmorphic features. Neurological examination showed central hypotonia and spastic diplegia, and contractures in lower limbs. Musculoskeletal examination showed scoliosis, while other systemic examinations were unremarkable.

Currently, at 15 years of age, she is having GDD, she can not walk and bound to wheel chair. She uses just single words to express her needs commands and was not enrolled in school or any academic rehabilitation centers due to her severe complications. Ophthalmology examination showed astigmatism. A skeletal survey showed severe thoracolumbar scoliotic deformity with convexity to the left side (Figure 1B). The affected individual (V-19) demonstrated congenital scoliosis with fused ribs (spondylocostal dysostosis), which resulted in the decrease in the total ribs cage capacity. Brain magnetic resonance imaging (MRI) bilateral symmetrical terminal myelination zones clustered cysts and abdomen ultrasound showed periportal fibrosis. She has three affected brothers with similar presentations such as GDD, mild-moderate ID, speech delay, and severe skeletal deformity such as scoliosis. The age of the three affected brothers (V-16, V-22, and V-24) was 10, 8, and 9 years (Table 1). The three affected brothers showed unremarkable echocardiogram examination, while the affected individual (V-19) revealed ASD. According to the weight parameters (SD and percentile), each affected individual had a common failure to thrive phenotype, respectively. Routine laboratory tests for all the four affected individuals (V-16, V-19, V-22, and V-24) revealed normal brain MRI, normal hearing, and ophthalmic examination (Table 1).

### Whole-Exome Sequencing and Sanger Segregation Analysis

A well-reputed company, CENTOGENE (Germany), performed the WES according to standard methods. After WES, as depicted by the family pedigree, variant filtration was performed based on the autosomal recessive pattern of inheritance. Screening of rare disease-causing homozygous and compound heterozygous variants was preferred (Umair et al., 2020). Initial screening was conducted in the genes classified in OMIM and HGMD. However, variants classified as pathogenic, likely pathogenic, and variant of unknown significance (VUS) according to ACMG were significant. A schema of the variant filtering and selection steps for identifying variants in the present study have been summarized in Figure 2.

We identified a homozygous missense variant [c.947A > G; p.(Asp316Gly)] (NM_015058.1) in exon 8 of the *VWA8* gene, located on chromosome 13q14.11, which segregated with the disease phenotype within the family and verified using Sanger sequencing. All the four affected individuals (V-16, V-19, V-22, and V-24) were homozygous for the identified variant, both parents (IV-3 and IV-4) and three normal siblings (V-17, V-20, and V-21) were heterozygous (carrier), while three siblings (V-14, V-15, and V-25) were wild type for the identified variant. The identified variant was screened in four affected and 10 normal family members and segregated well with the phenotype, thus giving a LOD score of 3.06 (Duzkale et al., 2013). The identified variant (c.947A > G) was found five times in the heterozygous state in the gnomAD database containing 125,748 human exome sequences and 15,708 whole-genome sequences with a minor allele frequency of 0.0000208/5 and six times observed in heterozygous state in TOPMED (0.000023/6). The variant (c.947A > G) was not reported in the homozygous state in any of the databases (1000 genomes, ExAC, EVS, and gnomAD) and was highly conserved across multiple species (Figure 1D). The identified variant was also screened in 2000+ Saudi exomes and was not observed in a homozygous state, suggesting that the variant is rare and disease causing.

### *In Silico* Analysis

The identified variant’s pathogenicity index was calculated using different online available tools and was considered disease-causing (Table 2). According to the American College of Medical Genetics and Genomics (ACMG) guidelines, the variant was classified as a variant of unknown significance (VUS; Class 3). The identified variant has been uploaded to the LOVD database.^7^

### Protein 3D Modeling

Herein, using *in silico* methodology for wild type and mutant, the 3D-structure of VWA8 was modeled *via* I-TASSER server. The predicted VWA8 structure had a good degree of accuracy, and the final refined model was assessed *via* different evaluation programs. 3D-models of wild-type and mutated VWA8 proteins [p.(Asp316Gly)] were predicted and evaluated using online structure analysis tools. Ramachandran plot indicated that approximately 91 and 93% of residues in the wild type and mutant structure lie in allowed regions of torsion angles, respectively. 3D structures were subjected to the Errat protein structure verification server, which provided an overall satisfactory quality factor of wild type and mutant structure model as 88 and 90%, respectively.

Our analysis revealed that Asp316 interacts with Phe313, Pro314, Ser317, and Ala320. Aspartic acid is a charged amino acid and often forms salt bridges, while glycine is the simplest amino acid with a single hydrogen atom as its side chain. Substitution of aspartic acid to glycine disturbs interaction with surrounding amino acid residues, and these new interactions, in turn, might potentially disrupt both protein secondary structure and function. Using DUET, ENCoM, and mCSM, we predicted that Asp316Gly mutation would cause a –0.792, 0.148, and –0.967 kcal/mole change ΔΔG, respectively, indicating that the mutation would greatly destabilize the protein structure and hence disrupt VWA8 function. VWA8^WT^ and VWA8^Trunc^ both were composed of helices, β-sheets, and coils; in KIAA0825^WT^, there were 23 α-helices, while KIAA0825^Trunc^ has 22 α-helices, along with 6 β-sheets reported in both structures (Figures 1E–H).

### Human and Zebrafish *vwa8* Shared the Consensus Sequence and Conserved Domain

The human and zebrafish *vwa8* shared high similarity and conserved domain and also have mitochondrial localization sequence. Zebrafish *vwa8* (Gene bank accession number NM_001128338.1) is located in chromosome 9 and has 45 exons. The human and zebrafish vwa8 proteins have high similarity. The online conserved domain search tool^8^ showed high amino acid similarity between the conserved VWA8 domain (Figure 3A).

To check whether zebrafish vwa8 is also localized to mitochondria, the amino acid sequence was searched using MitoProt II-v1.101. The zebrafish vwa8 contains 6 amino acids mitochondrial localization signals with a probability of 0.9775 (Figure 3B).

### Knockdown of *vwa8* Zebrafish Partially Mimicking the Human *VWA8* Mutant Patients

Two antisense oligo morpholinos, i.e., i1e2 and E2i2, were designed to target exon 2 of the *vwa8* zebrafish. Each of the morpholino was injected individually and also in combination. The antisense morpholino (ile2) injected embryos showed significant knockdown of vwa8 compared to the 2 bp mismatch control injected embryos (Figure 3C).

**FIGURE 4:**
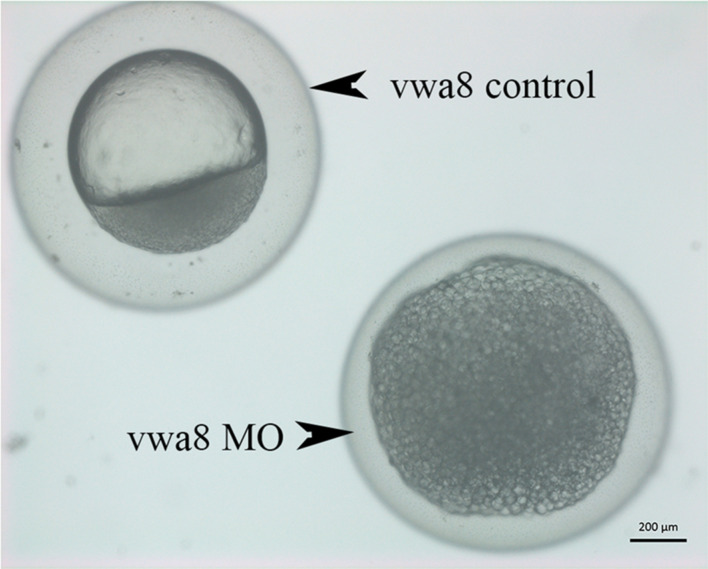
Zebrafish vwa8-i1e2 induced severe necrosis of embryos at the very early stage of zebrafish development. Representative micrograph of zebrafish embryos injected with either 5 base pair mismatch control morpholino (top) or with vwa8-i1e2 (bottom). Both morpholino was used at the concentration of 3 ng/embryo. The control embryos showed the sphere stage (4 hpf), whereas the zebrafish embryos injected with vwa8-i1e2 showed necrosis.

**FIGURE 5:**
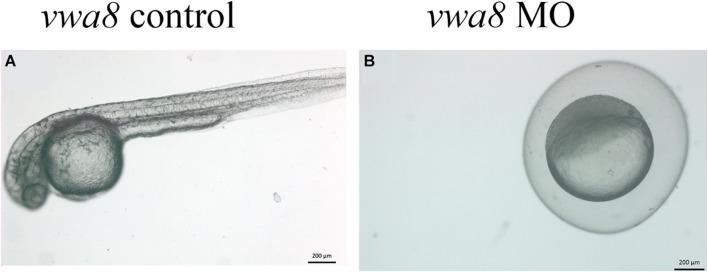
Zebrafish vwa8-i1e2 induced severe developmental delay. The figure shows representative photomicrograph taken at 24 h post-fertilization of wild-type zebrafish embryos. **(A)** Injected wither 5 bp mismatch i1e1 control. The control embryos developed normally up to 5 prim stage (24 hpf) and did not reveal any observable embryonic abnormalities. **(B)** 3 ng/embryo of vwa8-i1e2 antisense oligo morpholino. The vwa8-i1e2 morphants showed severe developmental delay by resembling spheres (4 hpf).

**FIGURE 6:**
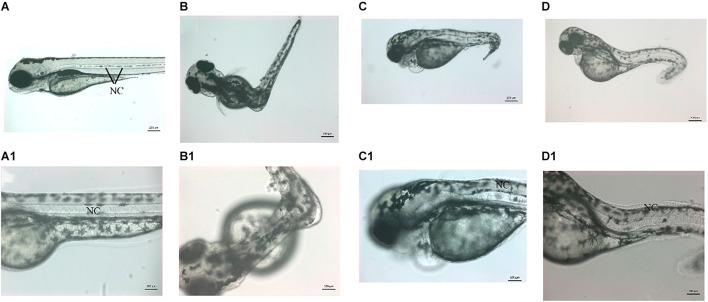
Knockdown of zebrafish vwa8 revealed severe scoliosis. Representative images of zebrafish embryos showing the whole embryos (top panel) and same embryos at high magnification (bottom penal) at 72 hpf. **(A,A1)** The control embryos developed normally and did not show any sign of toxicity and embryonic abnormality. The notochord (NC) is organized as a straight structure in control embryos shown by black lines. The vwa8-i1e2 morphants showed various degrees of scoliosis. **(B,B1)** Around 30% of the vwa8-i1e2 morphants (*n* = 250) had 90° curvature in the middle body; these embryos were also smaller than control embryos. The curvature of the notochord is more visible in the magnified image. **(C,C1,D,D1)** 20% of vwa8-i1e2 morphants (*n* = 250) showed bending of trunk region more toward posterior part, and disorganization of the notochord (NC) is quite evident in panel **(C1)**. The morphants were also of smaller size in total length as compared to control.

**FIGURE 7:**
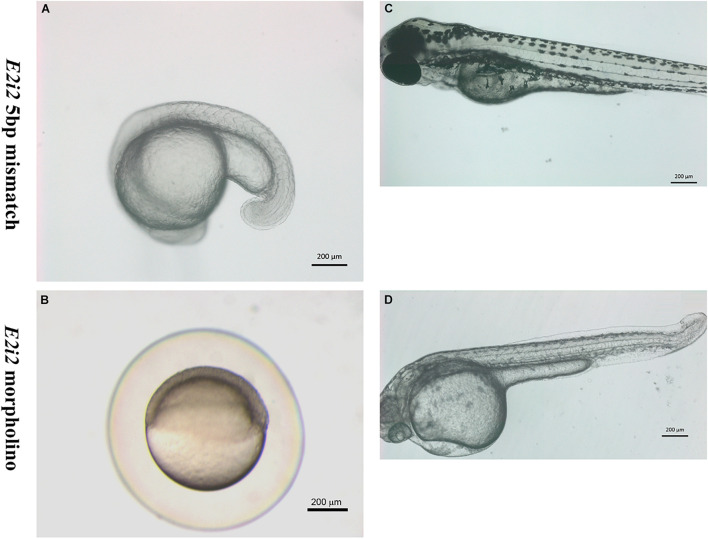
The splice morpholino vwa8-e2i2 induced less severe embryonic abnormalities. Representative micro-images of live zebrafish embryos injected with 5 bp mismatch control morpholino or vwa8-e2i2 (6 ng/embryo). **(A)** The control embryos were at the 19 somite stage (18 hpf). **(B)** The vwa8-e2i2 morphants showed developmental delay and were at 50% epiboly stage (6 hpf) simultaneously. **(C)** Control embryos at 72 hpf showed normal development and straight body, whereas **(D)** the morphants showed 24 h developmental delay and the curvature at the posterior end. The embryos were overall also smaller than the control.

**FIGURE 8:**
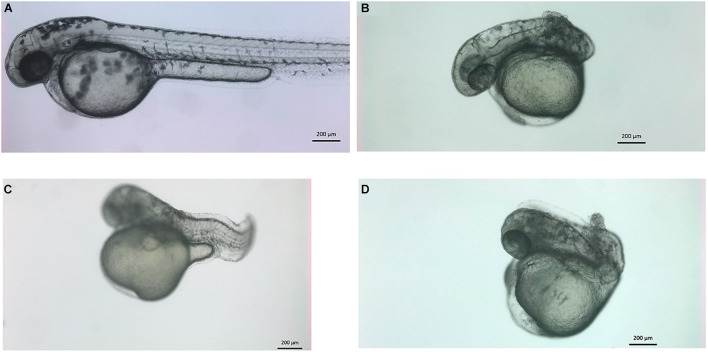
Knockdown of vwa8 resulted in the undeveloped posterior trunk and severe embryonic abnormalities. Representative images of zebrafish embryos **(A)** control (1:3 ratio of 5 base pair mismatch of vwa8-e2i2 and vwa8-i1e2 and **(B–D)** double knockdown 1 ng of vwa8-e2i2 and 3 ng vwa8-i1e2.

Various phenotypes were observed as a result of vwa8-i1e2 injections (Figures 4–8). As shown in Figures 5A,B, vwa8 splice morpholino 1 (i1e2; 3–5 ng/embryo) induced necrosis of embryos at a very early stage of embryonic development. The vwa8-i1e2 injected embryos developed up to 50% epiboly stage. However, more than 60% of these morphants died due to necrosis. On the contrary, the 5 base pair mismatch vwa8-i1e2 control did not show any toxic effects even at a concentration of 6 ng/embryos [control embryos: injected with 5 bp mismatch ile1 control].

### Zebrafish *vwa8* Morphants Displayed Developmental Delay

The vwa8-morphants exhibited severe developmental delay (Figures 5A,B). As shown in Figure 5A, the 5 bp mismatch control morpholino embryos reached 5 prim stages (24 hpf), whereas vwa8-i1e2 morphants showed developmental delay and resembled sphere stage (4 hpf).

### Zebrafish *vwa8* Morphants Exhibit Lack of Locomotion and Scoliosis

These morphants were smaller (size) than the control counterpart at a later stage (Figures 6B–D). The morphants were also not able to swim, suggesting a lack of locomotor activity. Among vwa8-i1e2 morphants, the majority of the survived embryos (60% of injected embryos and survived embryos) exhibited curved bodies “scoliosis” at 72 hpf (Figures 6B,B1). On close examination, it was noticed that the morphants had severe defects in the notochord. The notochord in zebrafish embryos is very much similar to the spinal cord in humans. The zebrafish vwa8-i1e2 morphants also exhibited heart defects and showed cardiac oedema and cardiac hypertrophy (Figures 6C,C1).

### Zebrafish *vwa8* Morphants Showed Disorganized Notochord

The second morpholino *vwa8*-e2i2, targeting the exon 2/intron 2 splice site of zebrafish *vwa8*, produced a less severe phenotype than *vwa8*-i1e2 morphants. No necrosis was observed in the injected embryos by injecting 6 ng/embryos (more than double of concentration compared to *vwa8*-i1e2). The *vwa8*-e2i2 morphants showed mild developmental delay. As shown, the 5 bp mismatch control *vwa8*-e2i2 injected embryos developed up to 19 somite stage (18 hpf), whereas the *vwa8*-e2i2 were at 50% epiboly stage (6 hpf) at the same time, which means the morphants were lagging at least 12 h in development as compared to control embryos (Figures 7A,B). The *vwa8*-e2i2 morphants continue to exhibit developmental delay at later stages. As shown in Figure 7C, the 5 bp mismatch control-injected embryos reached up to 72 hpf developmental stage, but *vwa8*-e2i2 morphants were at 48 hpf developmental stage. The *vwa8*-e2i2 morphants did not show severe defects in the body. Severe disorganization of notochord was observed at the posterior trunk (Figure 7D).

### Zebrafish *vwa8* Morphants Displayed Undeveloped Posterior Trunk and Severe Embryonic Abnormalities

The co-administration of *vwa8*-e2i2 and *vwa8*-i1e2 resulted in severe embryonic abnormalities in zebrafish embryos. The concentration of both morpholinos was optimized to reduce embryonic lethality. The best combination in which most of the injected embryos survived was in the ratio of 1:5 of *vwa8*-i1e2 and *vwa8*-e2i2, respectively. The zebrafish embryos at 72 hpf with various phenotypes resulting from the morpholinos’ co-administration are shown in Figures 8A–D. It is evident from these images that double knockout of zebrafish *vwa8* resulted from either complete loss of posterior trunk (Figures 8B,D) or severe disorganization of the notochord (Figure 8C).

## Discussion

Herein, we characterized a large consanguineous Saudi family segregating autosomal recessive complex developmental disorder. The affected individuals exhibited features such as global developmental delay, spastic diplegia, microcephaly, scoliosis, pneumonia, dyspnea, fever, progressive inability to walk, cardiovascular anomalies, brain atrophy, Achilles tendon contracture, lower limb hypertonia, limb hypertonia, hyperactive deep tendon reflexes, thoracic scoliosis, abnormality of the hip bone, and abnormality of the sphenoid sinus. The affected members have a variable degree of phenotypic severity. However, developmental delay, speech delay, scoliosis, and paralysis were shared among all the affected individuals.

Using DNA of the affected individuals, WES revealed a homozygous missense variant [c.947A > G; p.(Asp316Gly)] in the *VWA8* gene (Figure 1C). Segregation was confirmed using traditional bi-directional Sanger sequencing in all the available family members. The variant was present in exon 8 of the *VWA8* gene mapped on chromosome 13q14.1. *In silico* analysis and 3D homology modeling suggested the variant as disease-causing and might affect the secondary structure of the VWA8 protein. Further, the variant was not observed in homozygous state in different online publicly available databases such as gnomAD and ExAC. The identified variant [p.(Asp316Gly)] might result in loss of interactions with other essential proteins and might affect the protein function and downstream signaling. Zebrafish vwa8 morphants revealed several phenotypes such as developmental delay, cardiovascular anomalies (cardiac edema and cardiac hypertrophy), disorganized notochord, severe skeletal anomalies, lack of locomotion, and scoliosis.

The differential expression of *vwa8* mRNA at various zebrafish embryonic development stages indicates the stage-specific requirement of vwa8 in zebrafish. The presence of ZF *vwa8* mRNA in oocytes means that this gene has been maternally transcribed. A high percentage of necrosis of zebrafish embryo upon knockdown of vwa8 by anti-sense oligo morpholino has also been observed in this study, indicating that vwa8 is indispensable for early embryonic development in zebrafish. The exact molecular function, which vwa8 plays during zebrafish embryonic development, still needs to be explored. However, the expression of *vwa8* mRNA in oocytes reflects that it is one of those maternally transcribed genes. The ATPase activity is one of the functions attributed to vwa8 in humans, and high similarity between human and zebrafish vwa8 domains shows its conservation in many vertebrates. In frogs and fish, the first developmental asymmetry is established by forming the Balbiani body (Bb) composed of the mitochondrial germ plasm mRNAs (Kloc et al., 2004). It is most likely that the zebrafish *vwa8* could be among other maternally transcribed genes, which are necessary for mitochondrial function. Thus, in this context, the function of mitochondria and compromised activity should be checked in zebrafish vwa8 mutant or morphants.

So far, mouse model of the VWA8 mutant has not been developed. In order to create the vwa8 mutant model in animals, zebrafish (Danio rerio) was selected as it is much easier to induce genetic manipulation in zebrafish embryos as compared to other animal models. The zebrafish and human vwa8 genes are very much conserved, and the ATPase domain was also conserved; hence, two antisense morpholinos were designed to target the exon 2 of zebrafish vwa8 as the ATPase domain lie in exon 2. Using the two-splice modifying oligos in separate embryos and eliciting the same phenotype strongly supports the hypothesis that the phenotype observed is due to the knockdown of the targeted gene (Bill et al., 2009; Stainier et al., 2017). VWA8 is a mitochondrial and peroxisomal protein reported to play a key role in regulating ATPase activity inside the cell. Using P7 and P56, mouse tissues VWA8a and VWA8b were expressed in the cerebellum, left and right hemisphere, heart, lungs, spleen, and upper and lower limbs (Grewe et al., 2018). It has been observed that VWA8 possesses ATPase activity, suggesting its involvement in the energy-consuming biological processes in the body (Luo et al., 2019). Loss of *vwa8* using KO mouse liver cells produces a mitochondrial defect that may be sensed by NOX4, leading to a rise in ROS that results in higher hepatocyte nuclear factor 4 alpha (HNF4a). The compensatory HNF4a response results in higher oxidative capacity and even higher ROS production. Thus, VWA8 is suggested as an AAA+ ATPase protein that plays a role in mitochondrial protein quality (Luo et al., 2019).

The vwa8 mice knockout (IMPC^9^) revealed various types of skeletal anomalies, including digit abnormalities, scoliosis (spinal cord anomalies), and facial dysmorphism. In the knockout mice, the *vwa8* gene is not present, leading to different phenotypes compared to humans. Data from Allen Brain Atlas^10^ (*in situ* hybridization) also revealed a high level of vwa8 expression in the cerebellar granule cell layer.^11^ The presence of scoliosis phenotype in the *vwa8* knockout mice supported the notion that VWA8 pathogenesis might cause defects in skeletal development and associated abnormalities, thus supporting the data presented here.

Using genome-wide association studies, VWA8 has been associated with autism spectrum disorders (ASDs) (Cusco et al., 2009; Anney et al., 2010). Similarly, a study linked human Vwa8 SNPs comorbid migraine and bipolar disorder (Oedegaard et al., 2010). The KO Vwa8 was also associated with variations in the corpus callosum size, responsible for multiple behavioral and cognitive disorders in humans, such as schizophrenia, autism, attention deficit hyperactivity, and bipolar disorder (Newbury and Rosen, 2012). Proteins showing ATPase activities have been associated with a wide range of cellular processes, such as gene expression, DNA replication, membrane fusion, microtubule severing, protein degradation, signal transduction, and organelle biogenesis (Hanson and Whiteheart, 2005; Erzberger and Berger, 2006; Tucker and Sallai, 2007). Furthermore, ENCODE data suggested that Vwa8 is mainly expressed in the thymus and whole brain (ENCODE: 2004). Given the potential importance of this highly conserved protein in energy metabolism and pathologies, obtaining a comprehensive spatial and developmental expression pattern for Vwa8 is an essential first step toward a broader understanding of its function.

Recently, biallelic variants in the *VWA1* gene have been associated with recessive hereditary motor neuropathy (Deschauer et al., 2021; Pagnamenta et al., 2021). The family reported in the present study has several affected individuals with overlapping phenotypes as reported in the *VWA1* mutated patients. As both VWA1 and VWA8 belong to the same protein family, our data provide strong evidence that the VWA gene/protein family of extracellular matrix protein is associated with complex neurodevelopmental and skeletal disorders.

In conclusion, we report for the first time that intragenic variants in the *VWA8* gene might cause severe developmental and skeletal phenotypes in humans. Our zebrafish data strongly suggest the conserved function of vwa8 in humans and zebrafish supported by the observation of phenotypic hallmarks including developmental delay, microcephaly, and scoliosis in morpholino injected fish resulting in knockdown of vwa8. Nevertheless, more solid evidence of epigenetic and additional functional studies are required to investigate VWA8 functional aspects to uncover the pathophysiology resulting in human developmental phenotypes.

## Data Availability Statement

The datasets presented in this study can be found in online repositories. The names of the repository/repositories and accession number(s) can be found below: LOVD online database [https://www.lovd.nl/]. LOVD entry {Individual # 00377566}: https://databases.lovd.nl/shared/individuals/00377566.

## Ethics Statement

The studies involving human participants were reviewed and approved by KAIMRC. Written informed consent to participate in this study was provided by the participants’ legal guardian/next of kin. The animal study was reviewed and approved by Department of Zoology, King Saud University, Riyadh, Kingdom of Saudi Arabia. Written informed consent was obtained from the individual(s), and minor(s)’ legal guardian/next of kin, for the publication of any potentially identifiable images or data included in this article.

## Author Contributions

MU drafted the manuscript and analyzed the data. MAldr collected the samples, analyzed the clinical data, and performed the experiments. KA, MN, MAld, AA-R, and HA analyzed the genomic data. MF and MW performed the experiments on zebrafish and edited the manuscript. MAlf edited the manuscript and conception and design of the work. All authors contributed to the article and approved the submitted version.

## Conflict of Interest

The authors declare that the research was conducted in the absence of any commercial or financial relationships that could be construed as a potential conflict of interest.

## Publisher’s Note

All claims expressed in this article are solely those of the authors and do not necessarily represent those of their affiliated organizations, or those of the publisher, the editors and the reviewers. Any product that may be evaluated in this article, or claim that may be made by its manufacturer, is not guaranteed or endorsed by the publisher.

## Footnotes

1
http://genome.ucsc.edu/

2
http://frodo.wi.mit.edu/primer3/

3
https://gnomad.broadinstitute.org/

4
https://evs.gs.washington.edu/

5
https://www.genome.gov/27528684/1000-genomes-project

6
https://www.genome.jp/tools-bin/clustalw

7
https://databases.lovd.nl/shared/individuals/00377566

8
https://www.ncbi.nlm.nih.gov/Structure/cdd/wrpsb.cgi

9
https://www.mousephenotype.org/

10
www.brain-map.org

11
https://mouse.brain-map.org/gene/show/85497

## References

[B1] Anney R. Klei L. Pinto D. Regan R. Conroy J. Magalhaes T. R. (2010). A genome-wide scan for common alleles affecting risk for autism. *Hum. Mol. Genet.* 19 4072–4082. 10.1093/hmg/ddq307 20663923 PMC2947401

[B2] Badenhop R. F. Moses M. J. Scimone A. Mitchell P. B. Ewen-White K. R. Rosso A. (2002). A genome screen of 13 bipolar affective disorder pedigrees provides evidence for susceptibility loci on chromosome 3 as well as chromosomes 9, 13 and 19. *Mol. Psychiatry* 7 851–859. 12232778 10.1038/sj.mp.4001114

[B3] Bill B. R. Petzold A. M. Clark K. J. Schimmenti L. A. Ekker S. C. (2009). A primer for morpholino use in zebrafish. *Zebrafish* 6 69–77. 10.1089/zeb.2008.0555 19374550 PMC2776066

[B4] Chen Y. Lawless C. Gillespie C. S. Wu J. Boys R. J. Wilkinson D. J. (2010). CaliBayes and BASIS: integrated tools for the calibration, simulation and storage of biological simulation models. *Brief Bioinform.* 11 278–289. 10.1093/bib/bbp072 20056731

[B5] Cusco I. Medrano A. Gener B. Vilardell M. Gallastegui F. Villa O. (2009). Autismspecific copy number variants further implicate the phosphatidylinositol signaling pathway and the glutamatergic synapse in the etiology of the disorder. *Hum. Mol. Genet.* 18 1795–1804. 10.1093/hmg/ddp092 19246517 PMC2671988

[B6] Dai Y. (2000). Convergence properties of nonlinear conjugate gradient methods. *SIAM J. Optim.* 10 345–358. 10.1137/s1052623494268443

[B7] De Fusco M. Marconi R. Silvestri L. Atorino L. Rampoldi L. Morgante L. (2003). Haploinsufficiency of ATP1A2 encoding the Na+/K+ pump alpha2 subunit associated with familial hemiplegic migraine type 2. *Nat. Genet.* 33 192–196. 10.1038/ng1081 12539047

[B8] Deschauer M. Hengel H. Rupprich K. Kreiß M. Schlotter-Weigel B. Grimmel M. (2021). Bi-allelic truncating mutations in VWA1 cause neuromyopathy. *Brain* 144 574–583. 10.1093/brain/awaa418 33459760

[B9] Duzkale H. Shen J. McLaughlin H. Alfares A. Kelly M. A. Pugh T. J. (2013). A systematic approach to assessing the clinical significance of genetic variants. *Clin. Genet.* 84 453–463. 10.1111/cge.12257 24033266 PMC3995020

[B10] Emsley P. Lohkamp B. Scott W. G. Cowtan K. (2010). Features and development of coot. *Acta Crystallogr. D Biol. Crystallogr.* 66 486–501. 20383002 10.1107/S0907444910007493 PMC2852313

[B11] Erzberger J. P. Berger J. M. (2006). Evolutionary relationships and structural mechanisms of AAA+ proteins. *Annu. Rev. Biophys. Biomol. Struct.* 35 93–3114. 10.1146/annurev.biophys.35.040405.101933 16689629

[B12] Grewe B. S. Richmond J. E. Featherston D. E. (2018). The spatial and developmental expression of mouse Vwa8 (von Willebrand domain-containing protein 8). *Gene Exp. Patterns* 29 39–46. 10.1016/j.gep.2018.04.004 29660410

[B13] Hanson P. I. Whiteheart S. W. (2005). AAA+ proteins: have engine, will work. *Nat. Rev. Mol. Cell Biol.* 6 519–529. 10.1038/nrm1684 16072036

[B14] Källberg M. Wang H. Wang S. Peng J. Wang Z. Lu H. (2012). Template-based protein structure modeling using the RaptorX web server. *Nat. Protocols* 7:1511. 10.1038/nprot.2012.085 22814390 PMC4730388

[B15] Kloc M. Bilinski S. Etkin L. D. (2004). The Balbiani body and germ cell determinants: 150 years later. *Curr. Top. Dev. Biol.* 59 1–36. 10.1016/s0070-2153(04)59001-4 14975245

[B16] Luo M. Mengos A. E. Ma W. Finlayson J. Bustos R. Z. Zhu Y. X. (2017). Characterization of the novel protein KIAA0564 (Von Willebrand Domain-containing Protein 8). *Biochem. Biophys. Res. Commun*. 487 545–551. 10.1016/j.bbrc.2017.04.067 28414126 PMC5824621

[B17] Luo M. Willis W. T. Coletta D. K. Langlais P. R. Mengos A. Ma W. (2019). Deletion of the mitochondrial protein VWA8 induces oxidative stress and an HNF4α compensatory response in hepatocytes. *Biochemistry* 58 4983–4996. 10.1021/acs.biochem.9b00863 31702900 PMC8925280

[B18] Meng E. C. Pettersen E. F. Couch G. S. Huang C. C. Ferrin T. E. (2006). Tools for integrated sequence- structure analysis with UCSF chimera. *BMC Bioinformatics* 7:339. 10.1186/1471-2105-7-339 16836757 PMC1570152

[B19] Newbury A. J. Rosen G. D. (2012). Genetic, morphometric, and behavioral factors linked to the midsagittal area of the corpus callosum. *Front. Genet.* 3:91. 10.3389/fgene.2012.00091 22666227 PMC3364465

[B20] Oedegaard K. J. Greenwood T. A. Johansson S. Jacobsen K. K. Halmoy A. (2010). A genome-wide association study of bipolar disorder and comorbid migraine. *Gene Brain Behav.* 9 673–680. 10.1111/j.1601-183x.2010.00601.x 20528957 PMC2970709

[B21] Pagnamenta A. T. Kaiyrzhanov R. Zou Y. Da’as S. I. Maroofian R. Donkervoort S. (2021). An ancestral 10-bp repeat expansion in VWA1 causes recessive hereditary motor neuropathy. *Brain* 144 584–600. 10.1093/brain/awaa420 33559681 PMC8263055

[B22] StainierDYR, Erez Raz E. Nathan D. Lawson N. D. Stephen C. Ekker S. C. (2017). Guidelines for morpholino use in zebrafish. *PLoS Genet.* 13:e1007000. 10.1371/journal.pgen.1007000 29049395 PMC5648102

[B23] Strähle U. Scholz S. Geisler R. Greiner P. Hollert H. Rastegar S. (2012). Zebrafish embryos as an alternative to animal experiments–a commentary on the definition of the onset of protected life stages in animal welfare regulations. *Reprod. Toxicol.* 33 128–132. 10.1016/j.reprotox.2011.06.121 21726626

[B24] Tucker P. A. Sallai L. (2007). The AAA+ superfamily–a myriad of motions. *Curr. Opin. Struct. Biol.* 17 641–652. 10.1016/j.sbi.2007.09.012 18023171

[B25] Umair M. Ballow M. Asiri A. Alyafee Y. Al Tuwaijri A. Alhamoudi K. M. (2020). EMC10 homozygous variant identified in a family with global developmental delay, mild intellectual disability, and speech delay. *Clin. Genet.* 98 555–561. 10.1111/cge.13842 32869858 PMC7756316

[B26] Umair M. Hassan A. Jan A. Ahmad F. Imran M. Samman M. I. (2016). Homozygous sequence variants in the FKBP10 gene underlie osteogenesis imperfecta in consanguineous families. *J. Hum. Genet.* 61 207–213. 10.1038/jhg.2015.129 26538303

[B27] Wardi Y. (1988). A stochastic steepest-descent algorithm. *J. Optimiz Theory App.* 59 307–323. 10.1007/bf00938315

[B28] Whittaker C. A. Hynes R. O. (2002). Distribution and evolution of von willebrand/integrin a domains: widely dispersed domains with roles in cell adhesion and elsewhere. *Mol. Biol. Cell* 13 3369–3387. 10.1091/mbc.e02-05-0259 12388743 PMC129952

[B29] Yang J. Yan R. Roy A. Xu D. Poisson J. Zhang Y. (2015). The I-TASSER Suite: protein structure and function prediction. *Nat. Methods* 12 7–8. 10.1038/nmeth.3213 25549265 PMC4428668

